# QuickStats

**Published:** 2014-04-18

**Authors:** FROM THE NATIONAL CENTER FOR HEALTH STATISTICS

**Figure:**
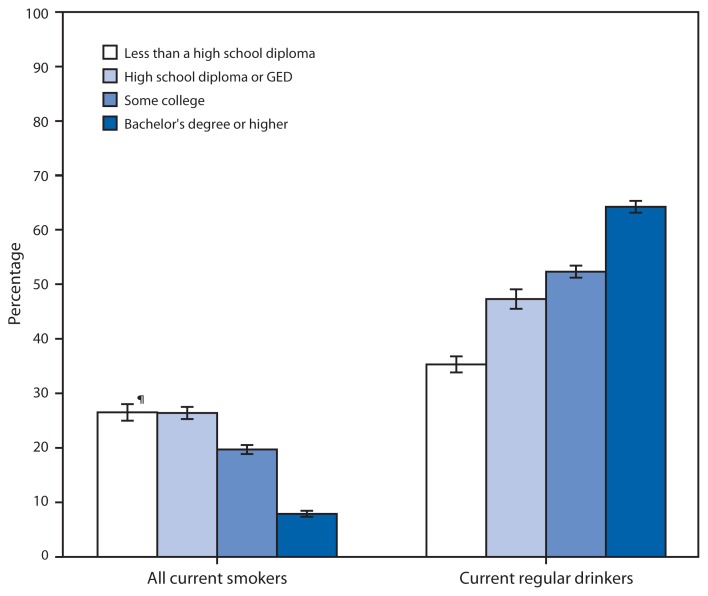
Percentage of Adults Aged ≥25 Years Who Were Current Smokers or Current Regular Drinkers,* by Education Level^†^ — National Health Interview Survey, United States, 2012^§^ **Abbreviation:** GED = general equivalency diploma. * Based on responses to separate questions that asked, “Have you smoked at least 100 cigarettes in your entire life?” Respondents answering “yes” were then asked, “Do you now smoke cigarettes every day, some days, or not at all?” Current smokers have smoked at least 100 cigarettes in their lifetime and currently smoke every day or some days. Respondents were also asked “In any 1 year, have you had at least 12 drinks of any type of alcoholic beverage?”; “In your entire life, have you had at least 12 drinks of any type of alcoholic beverage?”; and “In the past year, how often did you drink any type of alcoholic beverage?” A current regular drinker had at least 12 drinks in his or her lifetime and at least 12 drinks in the past year. ^†^Highest education completed consists of four categories: 1) adults with less than a high school diploma, 2) adults with a high school diploma or GED, 3) adults who attended some college including those receiving associate’s degrees (“some college”), and 4) adults who completed a bachelor’s degree or higher. ^§^Estimates are based on household interviews of a sample of the noninstitutionalized U.S. civilian population. Unknowns were not included in the denominators when calculating percentages. Percentages are age adjusted to the projected 2000 U.S. population as the standard population using four age groups: 18–44, 45–64, 65–74, and ≥75 years. ^¶^95% confidence interval.

Among adults aged ≥25 years in 2012, 26.5% of those who did not graduate from high school and 26.4% who had a high school diploma or GED were current smokers, compared with 19.7% who had attended some college and 7.9% with a college degree. In contrast, 64.2% of college graduates were current regular drinkers, compared with 52.3% of adults with some college, 47.3% of high school graduates or GED recipients, and 35.3% of adults who did not finish high school.

**Source:** Blackwell DL, Lucas JW, Clarke TC. Summary health statistics for U.S. adults: National Health Interview Survey, 2012 (provisional report). Vital Health Stat 2014;10(260). Available at http://www.cdc.gov/nchs/data/series/sr_10/sr10_260.pdf.

**Reported by:** Debra L. Blackwell, PhD, debra.blackwell@cdc.hhs.gov, 301-458-4103; Jacqueline W. Lucas, MPH; Tainya C. Clarke, PhD.

